# *N*-Benzyl-6-de­oxy-3,6-imino­methyl­ene-1,2,3,5-*O*-tetra­acetyl-α-d-1(*S*)-epiallo­furan­ose

**DOI:** 10.1107/S1600536811019702

**Published:** 2011-05-28

**Authors:** Qiurong Zhang, Chunli Wu, Pengyun Li, Weiyan Cheng, Hongmin Liu

**Affiliations:** a New Drug Reseach & Development Center, Zhengzhou Univresity, Zhengzhou 450001, People’s Republic of China

## Abstract

The mol­ecule of the title compound, C_22_H_27_NO_9_, an azasugar derivative, consists of one benzene ring and two fused rings, which have the *cis* arrangement at the ring junctions, and gives a V-shaped geometry. The inter­planar angle between the five- and six-membered rings is 65.69 (11)°. The crystal structure is stablized by weak inter­molecular C—H⋯O hydrogen bonds.

## Related literature

For the one-pot reaction used to obtain the title compound, see: Saito *et al.* (2002 ▶); Deshpandea *et al.* (2004 ▶). For the activity of aza­sugars, see: Compain *et al.* (2001 ▶, 2003 ▶). For their powerful inhibitory aptitude towards carbohydrate-processing enzymes, see: Guaragna *et al.* (2009 ▶).
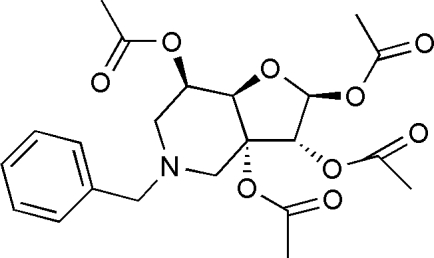


## Experimental

### 

#### Crystal data

C_22_H_27_NO_9_
*M* *_r_* = 449.45
Monoclinic, 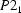

*a* = 8.1768 (16) Å
*b* = 9.0613 (18) Å
*c* = 15.591 (3) Å
β = 94.56 (3)°
*V* = 1151.5 (4) Å^3^
*Z* = 2
Mo *K*α radiation
μ = 0.10 mm^−1^
*T* = 291 K
0.20 × 0.18 × 0.16 mm

#### Data collection

Rigaku R-AXIS-IV diffractometer
Absorption correction: multi-scan (*SADABS*: Sheldrick, 1996 ▶) *T*_min_ = 0.980, *T*_max_ = 0.984
4341 measured reflections
2503 independent reflections
2173 reflections with *I* > 2σ(*I*)
*R*_int_ = 0.033

#### Refinement

*R*[*F* ^2^ > 2σ(*F* ^2^)] = 0.043
*wR*(*F* ^2^) = 0.107
*S* = 1.09
2503 reflections
290 parameters
1 restraint
H-atom parameters constrained
Δρ_max_ = 0.15 e Å^−3^
Δρ_min_ = −0.13 e Å^−3^

### 

Data collection: *R-AXIS II Software* (Rigaku, 1997 ▶); cell refinement: *R-AXIS II Software*; data reduction: *R-AXIS II Software*; program(s) used to solve structure: *SHELXS97* (Sheldrick, 2008 ▶); program(s) used to refine structure: *SHELXL97* (Sheldrick, 2008 ▶); molecular graphics: *SHELXTL* (Sheldrick, 2008) ▶; software used to prepare material for publication: *SHELXL97*.

## Supplementary Material

Crystal structure: contains datablocks I, global. DOI: 10.1107/S1600536811019702/zk2010sup1.cif

Structure factors: contains datablocks I. DOI: 10.1107/S1600536811019702/zk2010Isup2.hkl

Additional supplementary materials:  crystallographic information; 3D view; checkCIF report

## Figures and Tables

**Table 1:** Hydrogen-bond geometry (Å, °)

*D*—H⋯*A*	*D*—H	H⋯*A*	*D*⋯*A*	*D*—H⋯*A*
C15—H15*C*⋯O6^i^	0.96	2.33	3.161 (5)	144

Symmetry code: (i) 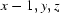.

**Fig. 1.**
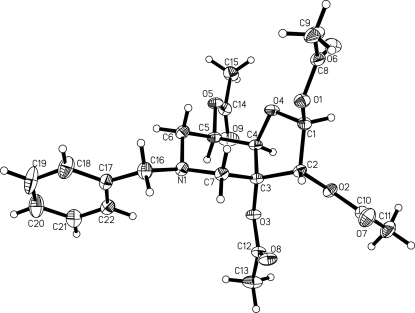
The molecular structure of the compound, with atom labels and 30% probability displacement ellipsoids for non-H atoms.

**Fig. 2.**
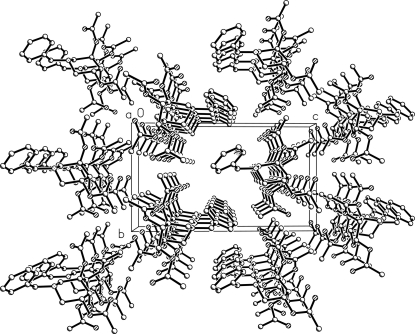
Packing of the molecules crystal structure of title compound, the molecules are connected by the C—H···O hydrogen bonds.

## supplementary crystallographic information

### Comment

Owing to their powerful inhibitory aptitude towards carbohydrate processing enzymes, azasugars undoubtedly represent one of the most attractive classes of carbohydrate mimetics (Guaragna *et al.*, 2009). As a contribution to the azasugars chemistry, we report here the crystal structure of the title compound, which was obtained under one-pot reaction (Sachin *et al.*, 2004 and Saito *et al.*, 2002) of *N*-benzyl-6-deoxy- 3,6-imino-methylene-1,2-*O*-isopropylidene-α-*D*-allofuranose.

In the crystal structure of the title compound (I) (Fig. 1), there are two fused rings (tetrahydrofuran ring and piperidine ring) having the *cis* arrangement at the ring junctions, giving a V-shaped molecule. The interplanar angle between the five and six membered rings is 65.69 (11)°. The torsion angles O1—C1—C2—C3, O1—C1—C2—O2 and C4—O4—C1—O1 around the carbon of hemiacetal group(C1) are 109.0 (2), -136.5 (2) and -137.1 (2)° respectively, which can confirm the hemiacetal group is β configuration. The molecules are linked into a framework by means of weak C—H···O hydrogen bonds (Table 1), one of whcih occurs between CH_3_ of C5-acetoxy moiety and O atoms of carbonyl of C1-acetoxy moiety, and three of which occur among CH and CH_2_groups of five and six membered rings and O atoms of carbonyl of three acetoxy moieties (Fig. 2).

### Experimental

*N*-benzyl-6-deoxy-3,6-imino-methylene- 1,2-*O*-isopropylidene-α-*D*-allofuranose (2.0 g, 6.2 mmol) was dissolved in 85% acetic acid (10 ml). A solution of 15% hydrochoric acid was added to this mixture. The resulting mixture was stirred for about 6 h at ambient temperature. After the material was consumed, the reaction mixture was evaporated under reduced pressure to dryness to yield yellow solid, which was directly used without purification. Acetic anhydride (5 ml) was added to the yellow solid in dry pyridine (5 ml). The mixture was stirred for about 5 h at ambient temperature. The reaction mixture was adjusted to neutral with saturated NaHCO_3_ under ice bath and filtered. The filtrate was extracted with EtOAc, dried (Na_2_SO_4_), and evaporated to obtained colorless oily. The oily was recrystallized from methanol to give the title compound as a white crystal. Crystals suitable for X-ray analysis were in two weeks by slow evaporation of methanol solution of the title compound at room temperature. ^1^H NMR (400 MHz, CDCl_3_) σ: 7.25–7.33 (5 H, m), 6.03 (1 H, s), 5.32 (1 H, s), 5.21 (1 H, m), 4.40 (1 H, d, *J* = 3.2 Hz), 3.62 (2 H, dd), 3.34 (1 H, d, *J*= 13.6 Hz), 2.81 (1 H, m), 2.54 (1 H, d, *J* = 13.6 Hz), 2.42 (1 H, m), 2.09 (3 H, s), 2.09(3 H, s), 2.08 (3 H, s), 2.07 (3 H, s), 2.04 (3 H, s); ^13^C NMR (100 MHz, CDCl_3_) σ: 170.2, 169.3, 168.9, 168.6, 137.0, 128.7, 128.3, 127.4, 99.3, 80.2, 77.4, 76.5, 68.1, 61.4, 51.4, 49.9, 21.1, 21.1, 21.0, 20.5.

### Refinement

All H atoms were placed geometrically and treated as riding on their parent atoms with C—H are 0.96 Å (methylene) or 0.93 Å (aromatic), 0.82 Å (hydroxyl)and *U*_iso_(H) =1.2*U*_eq_(C).

### Crystal data

**Table:**

C_22_H_27_NO_9_	*F*(000) = 476
*M**_r_* = 449.45	*D*_x_ = 1.296 Mg m^−^^3^
Monoclinic, *P*2_1_	Mo *K*α radiation, λ = 0.71073 Å
Hall symbol: P 2yb	Cell parameters from 399 reflections
*a* = 8.1768 (16) Å	θ = 2–25.1°
*b* = 9.0613 (18) Å	µ = 0.10 mm^−^^1^
*c* = 15.591 (3) Å	*T* = 291 K
β = 94.56 (3)°	PRISMATIC, colorless
*V* = 1151.5 (4) Å^3^	0.20 × 0.18 × 0.16 mm
*Z* = 2	

### Data collection

**Table:**

Rigaku R-AXIS-IV diffractometer	2503 independent reflections
Radiation source: fine-focus sealed tube	2173 reflections with *I* > 2σ(*I*)
graphite	*R*_int_ = 0.033
Detector resolution: 0 pixels mm^-1^	θ_max_ = 26.5°, θ_min_ = 1.3°
Oscillation frames scans	*h* = 0→10
Absorption correction: multi-scan (*SADABS*: Sheldrick, 1996)	*k* = −11→11
*T*_min_ = 0.980, *T*_max_ = 0.984	*l* = −19→19
4341 measured reflections	

### Refinement

**Table:**

Refinement on *F*^2^	Secondary atom site location: difference Fourier map
Least-squares matrix: full	Hydrogen site location: inferred from neighbouring sites
*R*[*F*^2^ > 2σ(*F*^2^)] = 0.043	H-atom parameters constrained
*wR*(*F*^2^) = 0.107	*w* = 1/[σ^2^(*F*_o_^2^) + (0.0659*P*)^2^] where *P* = (*F*_o_^2^ + 2*F*_c_^2^)/3
*S* = 1.09	(Δ/σ)_max_ < 0.001
2503 reflections	Δρ_max_ = 0.15 e Å^−^^3^
290 parameters	Δρ_min_ = −0.13 e Å^−^^3^
1 restraint	Extinction correction: *SHELXL*, Fc^*^=kFc[1+0.001xFc^2^λ^3^/sin(2θ)]^-1/4^
Primary atom site location: structure-invariant direct methods	Extinction coefficient: 0.040 (5)

### Special details

**Table:**

Geometry. All e.s.d.'s (except the e.s.d. in the dihedral angle between two l.s. planes) are estimated using the full covariance matrix. The cell e.s.d.'s are taken into account individually in the estimation of e.s.d.'s in distances, angles and torsion angles; correlations between e.s.d.'s in cell parameters are only used when they are defined by crystal symmetry. An approximate (isotropic) treatment of cell e.s.d.'s is used for estimating e.s.d.'s involving l.s. planes.
Refinement. Refinement of *F*^2^ against ALL reflections. The weighted *R*-factor *wR* and goodness of fit *S* are based on *F*^2^, conventional *R*-factors *R* are based on *F*, with *F* set to zero for negative *F*^2^. The threshold expression of *F*^2^ > σ(*F*^2^) is used only for calculating *R*-factors(gt) *etc*. and is not relevant to the choice of reflections for refinement. *R*-factors based on *F*^2^ are statistically about twice as large as those based on *F*, and *R*- factors based on ALL data will be even larger.

### Fractional atomic coordinates and isotropic or equivalent isotropic displacement parameters (Å^2^)

**Table:**

	*x*	*y*	*z*	*U*_iso_*/*U*_eq_	
N1	0.1159 (3)	0.9181 (3)	0.34251 (14)	0.0450 (6)	
O1	0.5766 (2)	1.1411 (2)	0.25616 (13)	0.0515 (5)	
O2	0.5092 (2)	0.8192 (2)	0.13055 (12)	0.0471 (5)	
O3	0.2300 (2)	0.7356 (2)	0.19908 (12)	0.0439 (5)	
O4	0.3209 (2)	1.1154 (2)	0.18522 (13)	0.0461 (5)	
O5	−0.0199 (2)	1.1524 (2)	0.15811 (13)	0.0489 (5)	
O6	0.5922 (3)	1.3316 (3)	0.16484 (19)	0.0771 (7)	
O7	0.7528 (3)	0.7358 (3)	0.18663 (16)	0.0700 (7)	
O8	0.4156 (3)	0.5948 (3)	0.27378 (14)	0.0589 (6)	
O9	−0.1360 (4)	1.0104 (3)	0.05304 (17)	0.0886 (9)	
C1	0.4843 (3)	1.0630 (3)	0.18939 (19)	0.0438 (6)	
H1A	0.5311	1.0805	0.1343	0.053*	
C2	0.4826 (3)	0.8971 (3)	0.20843 (16)	0.0393 (6)	
H2A	0.5621	0.8696	0.2561	0.047*	
C3	0.3042 (3)	0.8717 (3)	0.22978 (17)	0.0376 (6)	
C4	0.2149 (3)	0.9901 (3)	0.17496 (18)	0.0391 (6)	
H4A	0.2062	0.9591	0.1146	0.047*	
C5	0.0450 (3)	1.0225 (3)	0.20286 (18)	0.0428 (6)	
H5A	−0.0268	0.9385	0.1872	0.051*	
C6	0.0463 (3)	1.0483 (4)	0.29899 (19)	0.0486 (7)	
H6A	0.1118	1.1345	0.3153	0.058*	
H6B	−0.0645	1.0647	0.3149	0.058*	
C7	0.2889 (3)	0.8982 (3)	0.32508 (16)	0.0429 (6)	
H7A	0.3346	0.8149	0.3579	0.052*	
H7B	0.3509	0.9855	0.3433	0.052*	
C8	0.6243 (3)	1.2802 (4)	0.2351 (3)	0.0569 (8)	
C9	0.7193 (4)	1.3524 (5)	0.3088 (3)	0.0813 (12)	
H9A	0.7512	1.4497	0.2923	0.122*	
H9B	0.8155	1.2951	0.3251	0.122*	
H9C	0.6525	1.3590	0.3566	0.122*	
C10	0.6505 (3)	0.7422 (3)	0.1276 (2)	0.0496 (7)	
C11	0.6557 (5)	0.6680 (5)	0.0432 (2)	0.0733 (10)	
H11A	0.7565	0.6139	0.0420	0.110*	
H11B	0.6494	0.7407	−0.0018	0.110*	
H11C	0.5646	0.6013	0.0345	0.110*	
C12	0.2937 (3)	0.6055 (3)	0.2257 (2)	0.0473 (7)	
C13	0.1927 (5)	0.4817 (4)	0.1878 (3)	0.0770 (11)	
H13A	0.2399	0.3895	0.2075	0.115*	
H13B	0.1899	0.4862	0.1262	0.115*	
H13C	0.0831	0.4895	0.2054	0.115*	
C14	−0.1022 (4)	1.1307 (4)	0.08049 (19)	0.0501 (7)	
C15	−0.1374 (4)	1.2725 (4)	0.0348 (2)	0.0664 (10)	
H15A	−0.1958	1.2534	−0.0199	0.100*	
H15B	−0.0361	1.3219	0.0262	0.100*	
H15C	−0.2031	1.3339	0.0687	0.100*	
C16	0.0969 (4)	0.9206 (5)	0.43531 (19)	0.0587 (8)	
H16A	0.1255	1.0180	0.4576	0.070*	
H16B	0.1727	0.8504	0.4637	0.070*	
C17	−0.0747 (3)	0.8835 (4)	0.45673 (18)	0.0511 (8)	
C18	−0.1365 (5)	0.9410 (6)	0.5301 (2)	0.0842 (14)	
H18A	−0.0748	1.0086	0.5639	0.101*	
C19	−0.2898 (5)	0.8983 (8)	0.5533 (3)	0.107 (2)	
H19A	−0.3307	0.9381	0.6023	0.128*	
C20	−0.3806 (4)	0.7987 (7)	0.5048 (3)	0.0886 (15)	
H20A	−0.4820	0.7684	0.5215	0.106*	
C21	−0.3232 (4)	0.7438 (5)	0.4322 (2)	0.0690 (10)	
H21A	−0.3867	0.6778	0.3981	0.083*	
C22	−0.1714 (4)	0.7852 (4)	0.4087 (2)	0.0551 (8)	
H22A	−0.1332	0.7456	0.3590	0.066*	

### Atomic displacement parameters (Å^2^)

**Table:**

	*U*^11^	*U*^22^	*U*^33^	*U*^12^	*U*^13^	*U*^23^
N1	0.0465 (12)	0.0503 (14)	0.0377 (12)	0.0078 (11)	0.0004 (9)	0.0007 (11)
O1	0.0520 (11)	0.0460 (12)	0.0553 (12)	−0.0058 (10)	−0.0042 (8)	−0.0094 (10)
O2	0.0529 (10)	0.0469 (12)	0.0407 (10)	0.0098 (10)	−0.0010 (8)	−0.0082 (9)
O3	0.0525 (10)	0.0281 (9)	0.0493 (11)	0.0011 (8)	−0.0072 (8)	−0.0006 (9)
O4	0.0463 (10)	0.0301 (10)	0.0609 (12)	−0.0010 (8)	−0.0033 (8)	0.0031 (9)
O5	0.0541 (11)	0.0351 (10)	0.0551 (12)	0.0073 (9)	−0.0101 (9)	0.0059 (9)
O6	0.0976 (18)	0.0516 (15)	0.0848 (19)	−0.0140 (14)	0.0235 (14)	−0.0001 (15)
O7	0.0537 (11)	0.0827 (18)	0.0724 (15)	0.0187 (13)	−0.0030 (11)	−0.0070 (14)
O8	0.0694 (13)	0.0403 (12)	0.0643 (14)	0.0104 (10)	−0.0114 (11)	0.0051 (10)
O9	0.132 (2)	0.0629 (19)	0.0632 (16)	−0.0026 (16)	−0.0376 (15)	0.0019 (14)
C1	0.0451 (14)	0.0417 (16)	0.0437 (15)	−0.0028 (12)	−0.0019 (11)	−0.0016 (12)
C2	0.0458 (13)	0.0380 (15)	0.0333 (13)	0.0064 (11)	−0.0017 (10)	−0.0032 (11)
C3	0.0440 (13)	0.0284 (13)	0.0389 (14)	0.0011 (11)	−0.0059 (10)	−0.0008 (11)
C4	0.0478 (14)	0.0299 (14)	0.0382 (14)	−0.0005 (11)	−0.0048 (11)	−0.0011 (11)
C5	0.0453 (14)	0.0304 (14)	0.0505 (16)	0.0041 (12)	−0.0097 (12)	0.0031 (12)
C6	0.0478 (14)	0.0480 (18)	0.0493 (17)	0.0100 (13)	−0.0006 (12)	−0.0023 (14)
C7	0.0462 (13)	0.0450 (16)	0.0362 (13)	0.0053 (12)	−0.0052 (10)	0.0024 (13)
C8	0.0457 (15)	0.0437 (18)	0.082 (2)	−0.0031 (13)	0.0118 (15)	−0.0152 (17)
C9	0.0606 (19)	0.062 (2)	0.119 (3)	−0.0016 (18)	−0.0048 (19)	−0.040 (2)
C10	0.0489 (14)	0.0401 (15)	0.0604 (19)	0.0019 (13)	0.0086 (13)	0.0001 (15)
C11	0.084 (2)	0.070 (2)	0.068 (2)	0.012 (2)	0.0200 (17)	−0.020 (2)
C12	0.0549 (16)	0.0341 (15)	0.0523 (17)	0.0037 (13)	0.0014 (13)	0.0062 (13)
C13	0.086 (2)	0.042 (2)	0.099 (3)	−0.0069 (18)	−0.015 (2)	0.003 (2)
C14	0.0511 (15)	0.054 (2)	0.0439 (16)	0.0042 (14)	−0.0049 (12)	0.0069 (15)
C15	0.0665 (18)	0.068 (2)	0.064 (2)	0.0137 (17)	−0.0023 (15)	0.0268 (18)
C16	0.0624 (18)	0.074 (2)	0.0394 (16)	0.0000 (17)	0.0001 (13)	−0.0014 (16)
C17	0.0490 (15)	0.064 (2)	0.0404 (15)	0.0121 (15)	0.0019 (12)	0.0024 (15)
C18	0.074 (2)	0.117 (4)	0.061 (2)	0.002 (2)	0.0043 (18)	−0.034 (2)
C19	0.072 (2)	0.188 (6)	0.064 (2)	0.006 (3)	0.0209 (19)	−0.044 (3)
C20	0.0533 (17)	0.147 (5)	0.066 (2)	0.004 (3)	0.0058 (16)	−0.003 (3)
C21	0.0557 (17)	0.090 (3)	0.060 (2)	0.0025 (19)	−0.0044 (14)	0.001 (2)
C22	0.0602 (16)	0.060 (2)	0.0438 (16)	0.0102 (16)	−0.0012 (13)	−0.0006 (14)

### Geometric parameters (Å, °)

**Table:**

N1—C6	1.454 (4)	C8—C9	1.487 (5)
N1—C16	1.468 (4)	C9—H9A	0.9600
N1—C7	1.472 (3)	C9—H9B	0.9600
O1—C8	1.368 (4)	C9—H9C	0.9600
O1—C1	1.424 (3)	C10—C11	1.483 (5)
O2—C10	1.353 (3)	C11—H11A	0.9600
O2—C2	1.436 (3)	C11—H11B	0.9600
O3—C12	1.341 (3)	C11—H11C	0.9600
O3—C3	1.440 (3)	C12—C13	1.487 (5)
O4—C1	1.415 (3)	C13—H13A	0.9600
O4—C4	1.429 (3)	C13—H13B	0.9600
O5—C14	1.352 (4)	C13—H13C	0.9600
O5—C5	1.447 (3)	C14—C15	1.486 (5)
O6—C8	1.200 (4)	C15—H15A	0.9600
O7—C10	1.194 (4)	C15—H15B	0.9600
O8—C12	1.203 (3)	C15—H15C	0.9600
O9—C14	1.195 (4)	C16—C17	1.506 (4)
C1—C2	1.532 (4)	C16—H16A	0.9700
C1—H1A	0.9800	C16—H16B	0.9700
C2—C3	1.539 (4)	C17—C22	1.374 (4)
C2—H2A	0.9800	C17—C18	1.389 (5)
C3—C7	1.520 (4)	C18—C19	1.387 (6)
C3—C4	1.522 (4)	C18—H18A	0.9300
C4—C5	1.517 (4)	C19—C20	1.360 (7)
C4—H4A	0.9800	C19—H19A	0.9300
C5—C6	1.516 (4)	C20—C21	1.355 (6)
C5—H5A	0.9800	C20—H20A	0.9300
C6—H6A	0.9700	C21—C22	1.374 (5)
C6—H6B	0.9700	C21—H21A	0.9300
C7—H7A	0.9700	C22—H22A	0.9300
C7—H7B	0.9700		
			
C6—N1—C16	112.3 (2)	H9A—C9—H9B	109.5
C6—N1—C7	111.0 (2)	C8—C9—H9C	109.5
C16—N1—C7	111.2 (2)	H9A—C9—H9C	109.5
C8—O1—C1	115.3 (3)	H9B—C9—H9C	109.5
C10—O2—C2	118.0 (2)	O7—C10—O2	123.1 (3)
C12—O3—C3	120.49 (18)	O7—C10—C11	126.4 (3)
C1—O4—C4	107.5 (2)	O2—C10—C11	110.4 (3)
C14—O5—C5	116.8 (2)	C10—C11—H11A	109.5
O4—C1—O1	108.1 (2)	C10—C11—H11B	109.5
O4—C1—C2	108.4 (2)	H11A—C11—H11B	109.5
O1—C1—C2	111.0 (2)	C10—C11—H11C	109.5
O4—C1—H1A	109.8	H11A—C11—H11C	109.5
O1—C1—H1A	109.8	H11B—C11—H11C	109.5
C2—C1—H1A	109.8	O8—C12—O3	123.1 (3)
O2—C2—C1	108.3 (2)	O8—C12—C13	126.4 (3)
O2—C2—C3	108.6 (2)	O3—C12—C13	110.5 (3)
C1—C2—C3	102.2 (2)	C12—C13—H13A	109.5
O2—C2—H2A	112.4	C12—C13—H13B	109.5
C1—C2—H2A	112.4	H13A—C13—H13B	109.5
C3—C2—H2A	112.4	C12—C13—H13C	109.5
O3—C3—C7	113.3 (2)	H13A—C13—H13C	109.5
O3—C3—C4	104.26 (19)	H13B—C13—H13C	109.5
C7—C3—C4	111.4 (2)	O9—C14—O5	122.5 (3)
O3—C3—C2	116.0 (2)	O9—C14—C15	125.9 (3)
C7—C3—C2	110.0 (2)	O5—C14—C15	111.5 (3)
C4—C3—C2	101.2 (2)	C14—C15—H15A	109.5
O4—C4—C5	112.0 (2)	C14—C15—H15B	109.5
O4—C4—C3	103.7 (2)	H15A—C15—H15B	109.5
C5—C4—C3	112.6 (2)	C14—C15—H15C	109.5
O4—C4—H4A	109.4	H15A—C15—H15C	109.5
C5—C4—H4A	109.4	H15B—C15—H15C	109.5
C3—C4—H4A	109.4	N1—C16—C17	112.9 (2)
O5—C5—C6	108.9 (2)	N1—C16—H16A	109.0
O5—C5—C4	109.2 (2)	C17—C16—H16A	109.0
C6—C5—C4	112.3 (2)	N1—C16—H16B	109.0
O5—C5—H5A	108.8	C17—C16—H16B	109.0
C6—C5—H5A	108.8	H16A—C16—H16B	107.8
C4—C5—H5A	108.8	C22—C17—C18	117.5 (3)
N1—C6—C5	107.9 (2)	C22—C17—C16	122.0 (3)
N1—C6—H6A	110.1	C18—C17—C16	120.4 (3)
C5—C6—H6A	110.1	C19—C18—C17	120.4 (4)
N1—C6—H6B	110.1	C19—C18—H18A	119.8
C5—C6—H6B	110.1	C17—C18—H18A	119.8
H6A—C6—H6B	108.4	C20—C19—C18	120.3 (3)
N1—C7—C3	110.8 (2)	C20—C19—H19A	119.8
N1—C7—H7A	109.5	C18—C19—H19A	119.8
C3—C7—H7A	109.5	C21—C20—C19	119.9 (4)
N1—C7—H7B	109.5	C21—C20—H20A	120.0
C3—C7—H7B	109.5	C19—C20—H20A	120.0
H7A—C7—H7B	108.1	C20—C21—C22	120.2 (4)
O6—C8—O1	122.1 (3)	C20—C21—H21A	119.9
O6—C8—C9	126.7 (4)	C22—C21—H21A	119.9
O1—C8—C9	111.2 (4)	C17—C22—C21	121.7 (3)
C8—C9—H9A	109.5	C17—C22—H22A	119.2
C8—C9—H9B	109.5	C21—C22—H22A	119.2
			
C4—O4—C1—O1	−137.1 (2)	O4—C4—C5—C6	−67.2 (3)
C4—O4—C1—C2	−16.8 (3)	C3—C4—C5—C6	49.2 (3)
C8—O1—C1—O4	−78.8 (3)	C16—N1—C6—C5	−170.1 (2)
C8—O1—C1—C2	162.5 (2)	C7—N1—C6—C5	64.6 (3)
C10—O2—C2—C1	113.2 (3)	O5—C5—C6—N1	−178.5 (2)
C10—O2—C2—C3	−136.5 (2)	C4—C5—C6—N1	−57.5 (3)
O4—C1—C2—O2	105.0 (2)	C6—N1—C7—C3	−63.0 (3)
O1—C1—C2—O2	−136.5 (2)	C16—N1—C7—C3	171.1 (3)
O4—C1—C2—C3	−9.6 (3)	O3—C3—C7—N1	−65.2 (3)
O1—C1—C2—C3	109.0 (2)	C4—C3—C7—N1	51.9 (3)
C12—O3—C3—C7	−67.5 (3)	C2—C3—C7—N1	163.2 (2)
C12—O3—C3—C4	171.3 (2)	C1—O1—C8—O6	−1.0 (4)
C12—O3—C3—C2	61.0 (3)	C1—O1—C8—C9	179.9 (2)
O2—C2—C3—O3	27.8 (3)	C2—O2—C10—O7	0.3 (4)
C1—C2—C3—O3	142.1 (2)	C2—O2—C10—C11	179.3 (3)
O2—C2—C3—C7	157.9 (2)	C3—O3—C12—O8	−1.8 (4)
C1—C2—C3—C7	−87.8 (3)	C3—O3—C12—C13	177.9 (3)
O2—C2—C3—C4	−84.3 (2)	C5—O5—C14—O9	7.6 (5)
C1—C2—C3—C4	30.0 (2)	C5—O5—C14—C15	−170.2 (2)
C1—O4—C4—C5	158.4 (2)	C6—N1—C16—C17	76.4 (4)
C1—O4—C4—C3	36.7 (3)	C7—N1—C16—C17	−158.4 (3)
O3—C3—C4—O4	−161.9 (2)	N1—C16—C17—C22	33.4 (5)
C7—C3—C4—O4	75.6 (3)	N1—C16—C17—C18	−151.1 (4)
C2—C3—C4—O4	−41.2 (2)	C22—C17—C18—C19	0.5 (6)
O3—C3—C4—C5	76.7 (3)	C16—C17—C18—C19	−175.2 (4)
C7—C3—C4—C5	−45.7 (3)	C17—C18—C19—C20	0.6 (8)
C2—C3—C4—C5	−162.5 (2)	C18—C19—C20—C21	−1.8 (8)
C14—O5—C5—C6	−149.2 (2)	C19—C20—C21—C22	1.9 (7)
C14—O5—C5—C4	87.9 (3)	C18—C17—C22—C21	−0.5 (5)
O4—C4—C5—O5	53.6 (3)	C16—C17—C22—C21	175.2 (3)
C3—C4—C5—O5	170.1 (2)	C20—C21—C22—C17	−0.7 (6)

### Hydrogen-bond geometry (Å, °)

**Table:**

*D*—H···*A*	*D*—H	H···*A*	*D*···*A*	*D*—H···*A*
C2—H2A···O7	0.98	2.31	2.693 (3)	102
C5—H5A···O9	0.98	2.30	2.666 (4)	101
C7—H7A···O8	0.97	2.50	3.067 (4)	117
C15—H15C···O6^i^	0.96	2.33	3.161 (5)	144

Symmetry codes: (i) *x*−1, *y*, *z*.
